# Addressing professional burnout among Ukrainian medical workers during wartime: an online intervention study

**DOI:** 10.1192/j.eurpsy.2025.2382

**Published:** 2025-08-26

**Authors:** V. Rusanov, S. Lahutina, T. Abdriakhimova

**Affiliations:** 1 Bogomolets National Medical University, Kyiv, Ukraine; 2 Center for Affective Neuroscience, Charité - Universitätsmedizin, Berlin; 3 German National Academy of Sciences Leopoldina, Halle, Germany

## Abstract

**Introduction:**

Professional burnout among medical workers is a significant global concern characterized by emotional exhaustion, depersonalization, and a reduced sense of personal accomplishment. In Ukraine, the ongoing war has exacerbated this issue due to additional stressors such as exposure to traumatic events, resource limitations, and constant threats to personal safety. Medical professionals are facing unprecedented challenges, including increased patient loads from wartime casualties and psychological strain from working in high-risk environments.

**Objectives:**

Develop an online psychological training program to address professional burnout among Ukrainian medical workers during wartime, evaluate its feasibility in a conflict setting, and assess its preliminary effectiveness in reducing burnout levels and improving adaptive coping strategies.

**Methods:**

An online training was developed by specialists from Charité University Clinic (Berlin) and Bogomolets Medical University (Kyiv) under the “SOLOMIYA” project (https://solomiya.net.ua/).

Participants: 27 medical workers from Ukraine.

Study design was mixed-methods with quantitative assessments at three time points.

Intervention was delivered online via Zoom in Ukrainian to accommodate regional participants and wartime restrictions. Total duration was 4 hours over two 2-hour sessions, including psychoeducation, practical exercises, and interactive discussions.

Data collection scales: Maslach burnout inventory, Brief COPE, Professional Self-Efficacy, WHO-5 index, participant satisfaction survey.

**Results:**

Preliminary data from the 27 participants indicate a high level of satisfaction with the training program. The mean satisfaction score was 8.7 out of 10 (SD = 1.2), with scores ranging from 6 to 10 and a median of 9. The distribution of satisfaction scores was as follows: excellent (9–10): 59% (16 participants), very good (8): 15% (4), good (7): 19% (5), satisfactory (6): 7% (2).

These results suggest that the majority of participants found the training to be highly valuable and relevant to their needs. Participants reported that the exercises and coping strategies provided were useful for managing stress. Interactive discussions were noted as particularly helpful, demonstrating the importance of social support in reducing burnout. Due to the ongoing nature of data collection, comprehensive statistical analyses of changes in burnout levels, coping strategies, professional self-efficacy, and well-being are not yet available. However, initial observations indicate positive trends in these areas.

**Conclusions:**

Preliminary findings show that online psychological training is feasible and well-received by Ukrainian medical workers during wartime. High satisfaction scores suggest potential benefits. While definitive conclusions on effectiveness are premature, the positive reception aligns with research on tailored interventions in high-stress environments.

**Disclosure of Interest:**

None Declared

